# Applications of capillary action in drug delivery

**DOI:** 10.1016/j.isci.2021.102810

**Published:** 2021-07-01

**Authors:** Xiaosi Li, Yue Zhao, Chao Zhao

**Affiliations:** 1 Department of Chemical and Biological Engineering, The University of Alabama, Tuscaloosa, AL 35487, USA; 2 School of Software, Northwestern Polytechnical University, Taicang, Jiangsu 215400, China

**Keywords:** Drug delivery system, Pharmaceutical science, Materials science, Biomaterials

## Abstract

Contrary to the fact that capillary action is ubiquitous in our daily lives, its role in drug delivery has not attracted attention. Therefore, its application in medicine and disease treatment has not been actively developed. This perspective begins by reviewing the principles, advantages, and limitations of the three existing drug delivery strategies: non-covalent interaction, cavity loading, and covalent conjugation. Then, we discussed the principle of capillary action in drug delivery and the influencing factors that determine its performance. To illustrate the advantages of capillary action over existing drug delivery strategies and how the capillary action could potentially address the shortcomings of the existing drug delivery strategies, we described five examples of using capillary action to design drug delivery platforms for disease treatment: marker pen for topical and transdermal drug delivery, microneedle patch with a sponge container for pulsatile drug delivery, core-shell scaffold for sustained release of growth factors, oral bolus for insulin delivery to the esophagus, and semi-hollow floating ball for intravesical and gastroprotective drug delivery. Each of the five drug delivery platforms exhibits certain unique functions that existing drug delivery technologies cannot easily achieve, hence expected to solve specific practical medical problems that are not satisfactorily resolved. As people pay more attention to capillary action and develop more drug delivery platforms, more unique functions and characteristics of capillary action in drug delivery will be explored. Thus, capillary action could become an important choice for drug delivery systems to improve therapeutic drug efficacy, treat diseases, and improve human health.

## Graphical abstract

**Figure:**
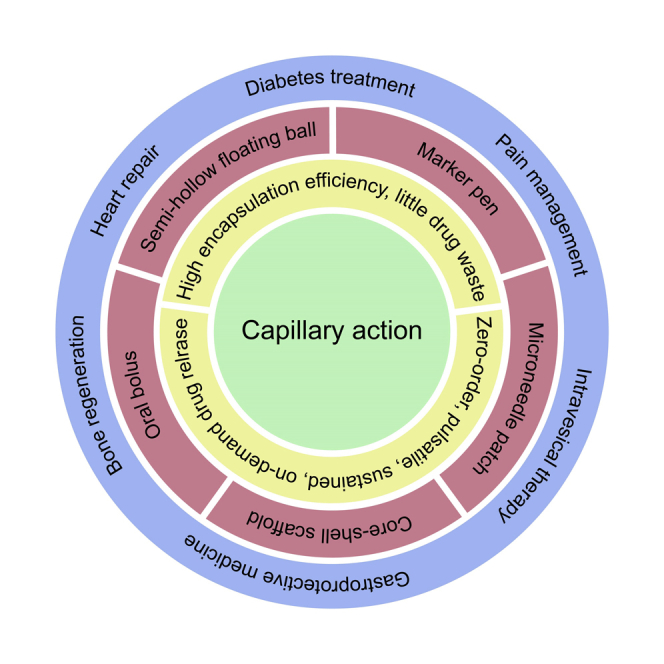

## Introduction

Drug delivery refers to approaches, formulations, technologies, and systems for transporting a drug in the body to maximize its therapeutic effect, reduce its side effects and improve patient convenience and compliance (Li et al., 2019; Tiwari et al., 2012; Wen et al., 2015). Drug delivery is approached by loading the drug in a carrier or medical device and releasing it in a controlled manner to the target area of the body. After decades of development since the 1950s, three major strategies were explored to obtain drug encapsulation and controlled release: non-covalent interaction, cavity loading, and covalent conjugation (Li et al., 2020; Lu et al., 2016; Wang et al., 2019; Zhao et al., 2013). Based on these strategies, numerous drug delivery devices, particles, and implants were reported, many of which were used clinically for disease treatment (Bulbake et al., 2017; Shomorony et al., 2019; Sorenson and Chesnut, 2019; van Eerden et al., 2020). Although progress was made, many clinical needs have not yet been met, and new strategies are always needed. In addition, the new strategy can provide options to replace or combine existing drug delivery strategies to overcome limitations and achieve functional synergy.

Here, we discussed the use of capillary action as a new strategy for drug delivery. Capillary action is present in many natural and technological processes (Mei et al., 2014; Mitropoulos, 2009), but few studies have examined it in drug delivery. Capillary action describes the interaction between liquid and material, which can encapsulate and control the release of drugs dissolved/dispersed in the liquid. Many drug delivery platforms with various structures and properties can be developed based on the principle of capillary action. These platforms may provide unique functions and characteristics that have not yet been implemented to meet clinical needs.

## Existing strategies for drug delivery

The existing drug loading and controlled release strategies can be divided into non-covalent interactions, cavity loading, and covalent conjugation.

### Non-covalent interactions

Non-covalent interactions are the most commonly used strategy in constructing drug delivery systems because of their diversity and universality and the advantages of simple preparation procedures (Wei et al., 2017). Drugs and materials in drug carriers interact through non-covalent interactions, such as hydrophobic interaction, electrostatic interaction, π-effect, hydrogen bonding, and van der Waals force, to achieve drug encapsulation and controlled release (Georgakilas et al., 2016; Karshikoff, 2006; Weldon et al., 2019). Among these non-covalent interactions, hydrophobic and electrostatic interactions are the most commonly used in drug delivery.

Hydrophobic interaction occurs by an attraction between the hydrophobic parts of the drug and the drug carrier. In the micro-emulsification process, polymers (e.g., poly [lactic-co-glycolic acid]; PLGA) or lipids and drugs are dissolved in a solvent and emulsified into droplets in a continuous aqueous phase (Jadhav et al., 2006; Kajbafvala and Salabat, 2017; Malik et al., 2012; Sah and Sah, 2015). The solvent is extracted from the polymer/lipid phase, the droplets solidify into nanoparticles/microspheres, and the drug is trapped in the nanoparticles/microspheres through hydrophobic interaction. Hydrophobic interaction is an attractive technique for encapsulating hydrophobic drugs because of the high drug encapsulation efficiency exceeding 90% (Ong et al., 2016). However, due to the weak interaction between hydrophilic molecules and hydrophobic carriers, the encapsulation efficiency of hydrophilic and/or amphiphilic small molecules is unsatisfactory (Li et al., 2020). In addition, this method involves using organic solvents in the manufacturing process, leading to solvent residue problems in the formulation (Sah and Sah, 2015; Shim and Sah, 2020).

Electrostatic interaction causes charged molecules to be adsorbed physically on oppositely charged carriers (Abraham et al., 2020; Kaasalainen et al., 2015; Kumar and Sharma, 2020; Liu et al., 2018; Mauri et al., 2017; Sims et al., 2020; Xiao et al., 2013; Zhao et al., 2012). This strategy has been widely used for gene delivery, where DNA and RNA are negatively charged and carriers positively charged (Nayerossadat et al., 2012; Sung and Kim, 2019; Zhang et al., 2016). Electrostatic interaction is advantageous due to its simple procedure for formulating the drug into the carrier by mixing them in a solution. However, this strategy only applies to charged molecules, and there are concerns about the cytotoxicity of positively charged materials (Fröhlich, 2012).

### Cavity loading

Cavity loading refers to the encapsulation of drugs in hollow carriers (Li et al., 2020; Shomorony et al., 2019; Wang et al., 2019). The hollow carrier has internal voids to provide a space for accommodating the drug and has a shell as a barrier to prevent the encapsulated drug from suddenly leaking out of the particles. Release involves passive diffusion of the drug across the shell or the active destruction of the shell. When modified with responsive molecules on the shell, the hollow particles release the incorporated drugs in response to external stimuli, such as light (Lin et al., 2013; Rwei et al., 2015; Timko et al., 2014; Zhan et al., 2016, 2017) and ultrasound (Rwei et al., 2017; Yan et al., 2013), that destroy the shell. This strategy is applicable to both hydrophobic and water-soluble drugs but particularly attractive for the latter. For example, liposomes and polymersomes have aqueous pockets that can entrap water, so the drugs dissolved/dispersed in water are encapsulated. The encapsulation efficiency can be as high as 40% (Rwei et al., 2015; Shomorony et al., 2019; Zhan et al., 2016, 2017). The limitation of this strategy is that the manufacturing procedure of hollow carriers is cumbersome, and therefore the formulation is relatively expensive (Liu et al., 2018; Rwei et al., 2015; Shomorony et al., 2019; Zhan et al., 2016, 2017). In addition, there are problems of undesirable drug leakage during storage and rapid initial release after administration leading to drug wastage, toxicity, and limited efficacy (Liu et al., 2018; Rwei et al., 2015; Shomorony et al., 2019; Zhan et al., 2016, 2017).

### Covalent conjugation

Covalent conjugation uses chemical linkers to immobilize drugs on large molecules (Zhang et al., 2020; Zhao et al., 2019). The synthesized compounds are called prodrugs, having little or no pharmacological activity but capable of releasing the active drug in the body by enzymatic reaction or chemical reaction or a combination of both (Rautio et al., 2018). The release rate can be tuned by adjusting the chemical linker type and the large molecules' chemical composition. For example, when a drug binds to a polymer through an ester bond, the drug is released by hydrolysis of the ester bond, and the hydrolysis rate can be reduced by increasing the hydrophobicity of the polymer chain (Zhao et al., 2019). Covalent bonds are more stable than non-covalent bonds; therefore, less drug is released prematurely, and sudden initial drug release is reduced. This strategy is highly dependent on the chemical structure of the drug. The drugs need to have appropriate functional groups so that they can be covalently bound to large molecules.

## Challenges in drug delivery

Although the existing drug delivery strategies are effective and widely used, there are still many challenges in practical applications, which create barriers between laboratory research and clinical translation, limit the improvement of drug efficiency, and increase the cost of formulations. The four most significant challenges are the biocompatibility of materials, insufficient drug encapsulation efficiency and the resulting drug waste, insufficient drug release control, and low drug bioavailability.

### Biocompatibility of materials

Biocompatibility is considered one of the essential criteria for drug carriers to be successfully used in drug delivery (Sundar et al., 2016). Many biocompatible polymers are approved by the U.S. Food and Drug Administration (FDA) for drug delivery application, such as polyethylene glycol, poly(lactic-co-glycolic acid) (PLGA), poly(glycolic acid) (PGA), and poly(lactic acid) (PLA). These polymers are non-toxic and can be eliminated from the body by natural metabolic pathways with minimal side effects (Marin et al., 2013). However, these FDA-approved materials cannot meet all clinical needs; hence copolymers based on them or other alternative materials with uncertain side effects have to be explored (Li et al., 2020; Zhao et al., 2020). In addition to the carrier itself, solvent residues in the carrier can also cause biocompatibility issues. Organic solvents are used usually in the manufacturing or processing of formulations, such as dissolving polymers for emulsification to prepare nanoparticles (Zhang et al., 2016), dissolving lipids to prepare liposomes through the film hydration method (Shomorony et al., 2019), and in the reactions to covalently bind drug to the large molecules (Zhang et al., 2020; Zhao et al., 2019). The solvents present in the final product must be below the limits set by the International Council for Harmonization of Technical Requirements for Pharmaceuticals for Human Use (ICH) as they are potentially harmful to those consuming these products. Moreover, removing and analyzing residual solvents from the product add additional costs.

### Insufficient drug encapsulation efficiency and drug waste

Encapsulation efficiency is the percentage of drugs successfully entrapped into the drug carriers. The separation and recovery of the unencapsulated drugs are usually not worth the loss. Therefore, unencapsulated drugs are discarded, resulting in drug wastage and increased costs. For water-soluble drugs, encapsulation efficiency achieved with liposomes is generally less than 40% (Nii and Ishii, 2005; Rwei et al., 2015; Shomorony et al., 2019; Zhan et al., 2016, 2017), which means that 60% of the drug is wasted. The encapsulation efficiency can exceed 90% for hydrophobic drugs but usually requires a higher material-to-drug ratio (Nii and Ishii, 2005).

### Insufficient drug release control

Conventional drug delivery systems release drugs in a "bell-shaped" concentration-time profile. The drug concentration increases to a peak and then decreases. The drug concentration is maintained for a relatively short period in the therapeutic window, and many drugs are released in ineffective or toxic concentrations. In addition to the "bell-shaped" concentration-time profile issue, many drug delivery systems have the problem of premature drug release before administration (Abdo et al., 2020; Shomorony et al., 2019). Immediately after formulation, the drug is released from the carrier, which continues during transportation and storage. The premature drug release leads to the depletion of the drug and shortens the formulation's shelf life, which may manifest as burst release and systemic toxicity, especially problematic with ultra-potent drugs (Kohane et al., 1998b; Shomorony et al., 2019).

Smart drug delivery systems, such as zero-order, pulsatile, and on-demand drug deliveries, are effective approaches that improve drug release control and provide more benefits than conventional drug delivery systems. Zero-order drug delivery refers to the process of a constant drug released per unit time from a carrier. It is particularly desired for potent drugs because it can precisely maintain constant plasma drug levels so that the drug's therapeutic effects can be maximized, and the toxic effects can be minimized (Zhao et al., 2017). Pulsatile drug delivery is defined as the rapid and transient release of a certain amount of molecules within a short time immediately after a predetermined off-released period, i.e., lag time (Jain et al., 2011). Pulsatile drug delivery can deliver a small portion of the total payload as a pulse so that the drug concentration can immediately increase above the therapeutic level to achieve its therapeutic effect but lower than the toxic level. In addition, the drug is exhausted, leaving little drug released in an ineffective concentration. On-demand drug delivery refers to the patients controlling the time, intensity, and duration of drug efficacy according to changing needs and conditions. There is no unwanted drug release in such a drug delivery system, and the drug is released from the carrier only when needed.

Despite the advantages over conventional drug delivery systems and the wide-ranging needs in many conditions, zero-order, pulsatile, and on-demand drug deliveries have not been used widely in clinical practice. Generally, the existing smart drug delivery systems are expensive because the drug carriers need to have some special structural designs or incorporate functional molecules to achieve smart drug delivery. In addition, the existing smart drug delivery systems are more research concepts than practical applications because they are only effective for a limited time. For example, Ma et al. reported a multi-pulse Parathyroid hormone (PTH) delivery device consisting of alternating drug and isolation layers (Dang et al., 2017). The isolation layer was made of poly-anhydride, which is biodegradable through surface erosion. The drug layer was made by mixing PTH and alginate, where alginate was used as a PTH carrier. The device achieved the pulsatile release of PTH after the isolation layer was removed through degradation, followed by PTH release from the drug layer. *In vitro*, the devices showed 21 pulses of PTH release over three weeks. Kohane et al. incorporated a sono-sensitizer into the shell of liposomes so that the liposomes are responsible for external stimulation (Rwei et al., 2017). After being triggered by ultrasound, the sono-sensitizer generates reactive oxygen species that react with the liposomal membrane, leading to the encapsulated drug release. After *in vivo* injection, this liposomal formulation achieved 6 times of triggered drug release. A new drug delivery strategy that makes it easier to achieve smart drug delivery and prolong its effectiveness will significantly promote drug delivery platform development and meet clinical needs.

### Low drug bioavailability for oral drug delivery system

Oral administration is the most commonly used route of drug administration because it is convenient, safe, and affordable (Bruno et al., 2013; Homayun et al., 2019; Renukuntla et al., 2013). However, oral drug delivery faces the challenge of low bioavailability. After oral administration, peptide and protein drugs are immediately degraded due to the gastrointestinal (GI) environment, resulting in low bioavailability. Even the drugs that can tolerate the GI environment, insufficient time for absorption in the target absorption site are another common cause of low bioavailability. An ideal oral drug delivery system should be able to (i) maintain the integrity of protein molecules until it reaches the absorption site; (ii) retain in the target absorption site and release the drug there (Renukuntla et al., 2013) to improve its bioavailability. In the past few decades, many technologies explored the possibility to increase the bioavailability of oral drug delivery systems; however, with limited success (Renukuntla et al., 2013).

## Capillary action in drug delivery

### Principle of capillary action in drug loading and controlled release

#### Capillary action retains the liquid to encapsulate and store drugs

**Figure 1:**
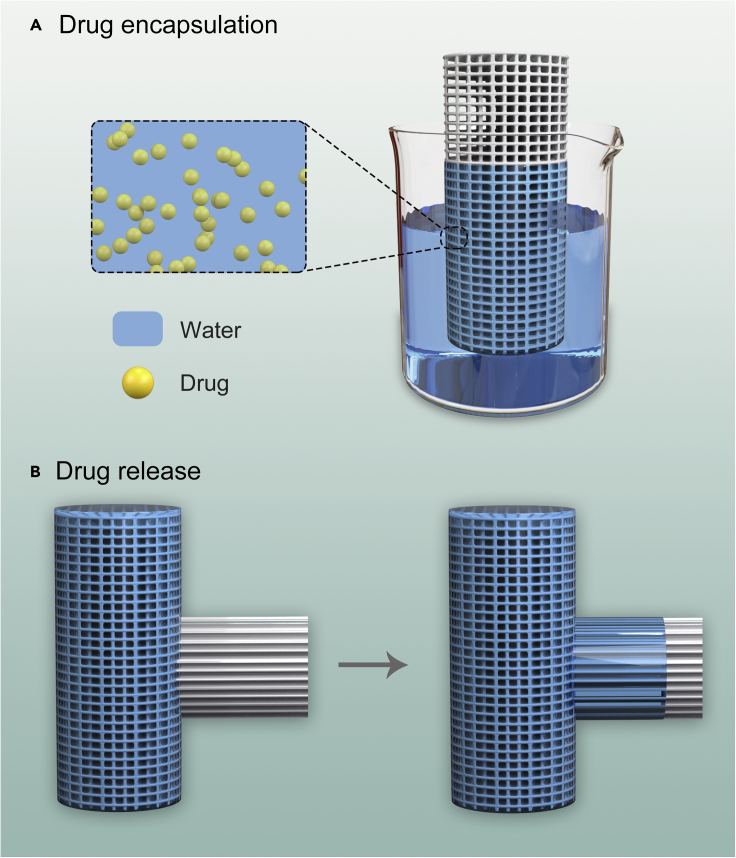
Principles of capillary action in drug loading and controlled release (A) Capillary action retains liquid to encapsulate and store drugs. (B) Capillary action transfers the liquid to deliver and release drugs.

Capillary action retains the liquid in the materials. For example, paper towels and sponges absorb liquid against gravity, and thin glass tubes can hold a small amount of water. When the liquid is enclosed, the drug dissolved/dispersed in the liquid is encapsulated in the materials (Figure 1A).

#### Capillary action transfers the liquid to deliver and release drugs

Capillary action transfers the liquid through the materials. For example, groundwater is transferred from the wet soil area to the dry area. Trees absorb water and get water to all the branches and leaves. When performing capillary blood sampling, blood is collected from the puncture site of the finger. When the liquid is transferred through the materials, the drug dissolved/dispersed in the liquid is transported simultaneously, thereby achieving the delivery and release of the drug at the target location (Figure 1B).

### Factors affecting the roles of capillary action in drug loading and controlled release

Capillary action results from competition between the forces of adhesion (liquid molecules are attracted and stick to other substances), cohesion (liquid molecules stay close together), and surface tension. Therefore, the factors affecting these forces determine the roles of capillary action in drug loading and its controlled release.

#### Surface-to-volume ratio of the material

Generally, the larger the surface area, the more liquid adhere to the surface. Thus, the surface-to-volume ratio should be proportional to the loading capacity of the material. Besides, the drug release is delayed when the surface-to-volume ratio is high because the liquid needs to pass a longer surface before entering the environment. The material can be made into a sponge-like porous structure or fiber bundles to increase the surface area. In addition, the diameter and number of pores or fibers can be adjusted to control drug loading and release from the material.

#### The polarity of the surface

Surface polarity significantly affects the adhesive force between the liquid and materials. Water molecules are more likely to attract hydrophilic or charged surfaces because these surface molecules are more polar than water molecules. Surface polarity can help the material absorb liquids to encapsulate the drugs. Besides, surface polarity can accelerate the release of the drug because the liquid is easier to wet and pass through the surface.

#### Nature of the liquid

The density and composition of the liquid determine the capillary action between the liquid and material. The greater the liquid density, the more difficult it is to encapsulate in the material, and the easier it is to release from the material due to gravity. The type and concentration of the solute in the liquid affect the capillary action because the presence of solutes changes the liquid density, forces of adhesion, cohesion, and surface tension of the liquid. Therefore, the loading and release of the drug from the material can be regulated by manipulating the nature of the liquid.

#### Temperature

The effect of temperature is important and complicated because its impact simultaneously affects forces of adhesion, cohesion, and surface tension. The degree of this change depends on the nature of the liquid and surface characteristics of the materials. The effect of temperature on drug loading and release by capillary action needs to be studied under different circumstances. The temperature effect allows the release of the drug from the material, triggered by changing the temperature.

### Predictive design of drug delivery platforms based on capillary action

Drug delivery platforms with various structural and functional characteristics can be developed based on capillary action in drug loading and controlled release. In this perspective, we discussed five predictive designs of drug delivery platforms. Each platform exhibits some unique characteristics and functions that existing drug delivery strategies cannot easily achieve. From these examples, capillary action has shown potential as a new drug delivery strategy to solve current challenges. The marker pen, microneedle patch with a sponge container, and core-shell scaffold improve the drug encapsulation and control drug release. The oral bolus and semi-hollow floating ball improve the drug availability of the oral drug.

#### Marker pen for high drug encapsulation and zero-order drug release

**Figure 2:**
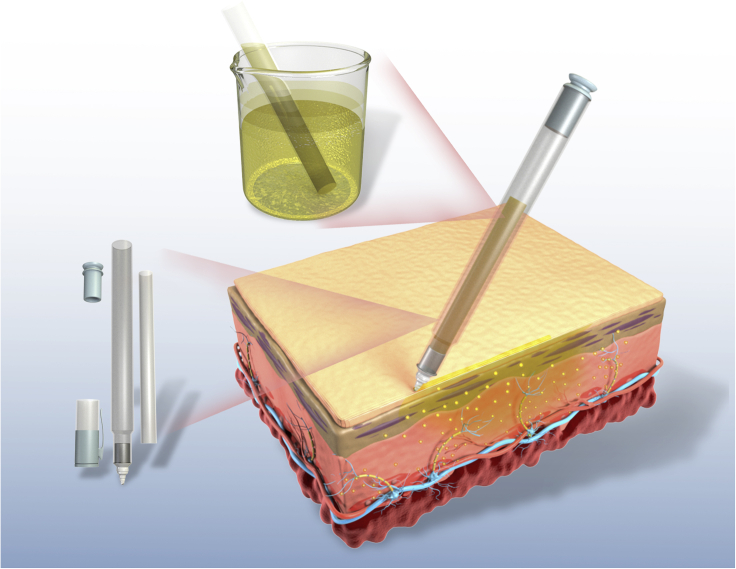
A schematic diagram of the marker pen as a platform for drug encapsulation and topical and transdermal drug delivery The pen core encapsulates the drugs by absorbing the drug solution. The encapsulated drug can be released by crossing the pen tip on the skin.

A marker pen is one of the most commonly used items in our daily lives. It stores ink in a sponge core through capillary action, and when the pen tip is moved across the surface of the substrate, it releases a constant amount of ink for writing. In addition, there is a cap to prevent the ink from drying out to have a long shelf life for a proper function. These features of the marker pen make it an ideal platform for drug delivery: (i) High drug encapsulation efficiency with no drug wastage. The drug can be easily encapsulated by immersing the spongy pen core in the drug solution (Figure 2). The drug solution automatically fills the pen core through the capillary action between the material and water. The marker pen can achieve 100% drug utilization because the drug can be reused until all the solution is absorbed. In addition, drug loading in the pen core can easily be controlled by adjusting the drug concentration in the solution; (ii) Zero-order drug release. There is no undesirable drug leakage during storage since the pen core retains the drug via capillary action. The marker pen can achieve linear drug release with respect to the length of the drawn line and the drug concentration of the solution, which is highly desired for improving drug efficacy and reducing its side effects (Li et al., 2021). Moreover, the drug concentration in the solution can be as low as possible so that the drug release from the pen tip can be precisely controlled at nanogram level or lower; (iii) Drug combination. The marker pen can be used for the co-delivery of multiple drugs by dissolving multiple drugs in one solution. Similarly, the marker pen can also be used to deliver many other ingredients along with the drug, such as chemical permeation enhancers (CPEs) that help drugs flow across biological membranes.

Two practical applications of the marker pen platform are the release of local anesthetics for pain management and antibiotics for local skin infections.

##### Use a marker pen to tame tetrodotoxin (TTX) for local anesthesia

The local anesthetic can be filled into the pen and applied to the target location by moving the pen tip on its surface. In addition to conventional local anesthetics, such as bupivacaine and tetracaine, the marker pen is suitable for controlling the release of potent neurotoxin-based local anesthetics.

TTX, a highly potent neurotoxin-based local anesthetic, binds to the voltage-gated sodium channels on nerve cell membranes and prevents sodium ions from entering neurons, thereby inhibiting the firing of action potentials of neurons (Kohane et al., 1998b). The effectiveness of TTX as a nerve block is approximately 1000 times that of conventional local anesthetics. Therefore, a very small TTX dose is required to achieve local anesthesia. Despite its ultra-potency, TTX has not yet achieved clinical use as a local anesthetic due to the considerable systemic toxicity. Besides, TTX has a very narrow therapeutic window (e.g., there is a small difference between the toxic and therapeutic levels of TTX for blocking nerves). For example, a single injection of 4 μg TTX in phosphate-buffered saline (PBS, pH 7.4) can produce sensory sciatic nerve block in rats for 2 hr and a single injection of 5 μg TTX is fatal to rats (Liu et al., 2018; Zhao et al., 2019).

The pharmacokinetics and bio-distribution profile of TTX can be improved by encapsulating TTX in delivery systems that allow sustained release. In the past two decades, drug-controlled release strategies were documented to obtain the encapsulation and controlled release of TTX. (i) Physical encapsulation in liposomes (Shomorony et al., 2019). Liposomes, core-shell structured hollow particles, can efficiently encapsulate TTX into the aqueous pockets with a relatively high loading efficiency. In addition, the hydrophobic shells prevent leakage of TTX, achieving sustained release. (ii) Physical adsorption through electrostatic interaction refers to the physical entrapment of negatively charged TTX molecules in positively charged carriers (Liu et al., 2018). (iii) Covalent conjugation. TTX is covalently bonded to the polymer backbone via the hydrolyzable bond (Zhao et al., 2019) and released via the hydrolysis of the chemical bond. The release rate can be tuned by adjusting the hydrophobicity of the chemical bond. Although these three strategies can efficiently encapsulate TTX, they release TTX in a “bell-shaped” concentration-time profile. The TTX concentration increases to a peak and then declines rapidly. TTX concentration is only maintained for a relatively short period in the therapeutic window, while a large portion of TTX is released at ineffective or toxic concentrations. The “bell-shaped” TTX release may manifest as burst release and systemic toxicity, limiting the initial dosing and therapeutic effect duration of TTX.

The marker pen platform is an ideal platform to tame TTX for local anesthesia. Similar to releasing a constant amount of ink onto paper, the marker pen can release a constant amount of TTX onto the tissue surface. In addition, the TTX concentration in the aqueous solution can be infinitely diluted to release only desired small amount of TTX. Thus, the marker pen can maintain the local concentration of TTX higher than the effective concentration but lower than the toxic level.

TTX-filled marker pen can be used as a local anesthetic for chronic skin pain management in patients with skin disorders. The patient or clinician can use the pen to apply TTX on the skin surface that needs pain relief. It may be necessary to deliver the CPEs along with TTX in pen to improve the permeability of TTX through the skin. A TTX-filled marker pen is a safe and on-demand approach to relieve local pain, allowing patients to control the time, intensity, and duration of anesthesia according to their changing needs and conditions. Thus, greatly improving patients' quality of life with chronic skin pain and reducing or obviating opioids use.

##### Marker pen for topical antibiotics

The marker pen filled with antibiotics can be used as topical antibiotics to treat many skin infections. An antibiotic-filled pen could improve compliance, minimize side effects, and prevent the systemic distribution of antibiotics. For example, the current treatment of children's acute otitis media (OM) includes a 10-day course of broad-spectrum oral antibiotics (Yang et al., 2016). However, the systemic administration of antibiotics usually causes side effects, including diarrhea, dermatitis, vomiting, and oral thrush (Hoberman et al., 2011). Antibiotics clinically used to treat OM can be dissolved in an aqueous solution and filled into the marker pen. The marker pen can be directly applied to the tympanic membrane through the external auditory canal. CPEs can be co-delivered together with the antibiotic using the marker pen to assist the flux of the antibiotic across the membrane (Yang et al., 2016).

#### Microneedle patch with a sponge container for pulsatile drug delivery

Intensive research has been conducted on microneedle patches as a therapeutic and vaccine delivery platform for transdermal drug delivery (Kim et al., 2012; Li et al., 2017). Microneedles can help drugs pass through the skin by creating micron-scale pathways. Most microneedles allow immediate or sustained drug release, where the drug is released in a relatively monotonic manner, and the drug concentration decreases over time. Pulsatile drug delivery allows repeated transient release of a certain amount of drug, an ideal complement to the microneedle patch technology to improve its therapeutic effect.

**Figure 3:**
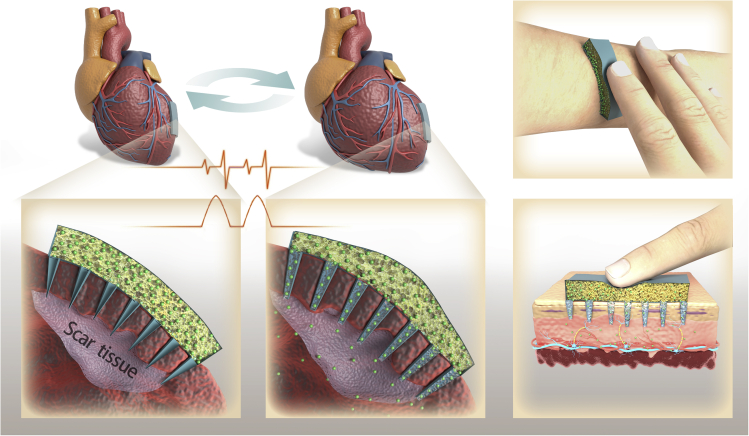
A schematic diagram of the microneedle patch with a sponge container to achieve pulsatile drug delivery for cardiac repair after acute myocardial infarction (MI) and on-demand local anesthesia

We introduced the microneedle patch with a sponge container to realize the pulsatile drug delivery of the microneedle patch (Figure 3). The sponge container is composed of fibers/pores, which retain the drug dispersion or solution by capillary action. The microneedles are hollow and connected to the container. When an external force is applied to the sponge container, the drug dispersion or solution will be injected into the target area through the hollow microneedles; thus, completing a pulse release of the drug. Two possible practical applications of a microneedle patch are cardiac repair after acute myocardial infarction (MI) and on-demand local anesthesia.

##### Microneedle patch for cardiac repair

Drug delivery systems have been used to deliver growth factors (GFs) for therapeutic heart regeneration after acute MI. GFs have an initial burst release for most drug delivery systems, and the release drops rapidly thereafter. The lack of appropriate spatial-temporal control of the drug release after transplantation or injection limits the therapeutic effect of GF. In addition, the potential toxicity of a high GF concentration may cause safety and cost-effectiveness issues (Wang et al., 2017).

Microneedle patch with a sponge container can realize the heart rhythm-induced pulsatile GF delivery to keep GF concentration stable for a long time. The GF solution can be filled in the sponge container. In each heart contraction cycle, the sponge container is stretched and compressed, pushing the GF solution through the microneedles to the injured area, thereby completing a pulse drug release.

The microneedles are designed to be gradient to achieve long-term stable pulsatile GF delivery. Certain microneedles can be blocked initially to prevent the release of high GF doses in the early stage. As the amount of GF solution in the container decreases, more microneedles will open due to the dissolution of the plug. Several microneedles connected will supplement the reduction in GF solution in the container so that the level of GF flowing into the injured site remains stable. The sponge container can also be filled with cardiac stromal cells to achieve long-term stable pulsatile GF delivery, which can continuously secret GFs in a stable amount (Tang et al., 2018).

##### Microneedle patch for on-demand local anesthesia

Locally injected anesthetics, such as bupivacaine, chloroprocaine, lidocaine, procaine, and tetracaine, are effective in pain relief(Bagshaw et al., 2015). They block the local nerve and numb the innervated area by that nerve; however, they do not last long. Multiple high-frequency local anesthetics injections are needed to manage acute and chronic pain, which increases the patient's compliance. Many investigators developed drug delivery systems to provide a prolonged duration of local anesthesia, lasting days to approximately one week from a single injection (Kohane et al., 1998a). However, the drug release from these formulations is monotonous, and once established, the nerve block cannot adapt to the patient's changing conditions.

Long-lasting and safe local anesthetics that enable patients to adjust the degree of local analgesia according to their changing needs and conditions would be beneficial. Microneedle patches with a sponge container can be used for transdermal delivery of local anesthetics for on-demand local anesthesia. The local anesthetics can be filled and stored in the sponge container. The volume of the container is adjustable to realize highly flexible drug loading for short-term or long-term use. When embedded in a muscle for transdermal administration, the patient can press the container to achieve the pulsatile release of the local anesthetics through the microneedle. Thus, this device enables patients to adjust the strength of their fingers based on their real-time feelings to control the drug release.

#### Core-shell scaffold for sustained release of GF

Bone morphogenetic proteins (BMPs) are multi-functional GFs that play a vital role in bone growth, maturation, and regulation (Blackwood et al., 2012; Oliveira et al., 2021). The most common clinical technique using BMPs is a relatively straightforward method of immersing the collagen scaffold in GF solution before implantation (Blackwood et al., 2012). Although this technique has been clinically accepted, it exhibits poor GF release, rapidly elutes, and presents BMP at the wound site with a concentration much higher than found in normal physiological conditions. It would be beneficial if the BMP release from the scaffold could be efficiently controlled and BMP concentration was maintained at an appropriate physiological level during bone regeneration.

**Figure 4:**
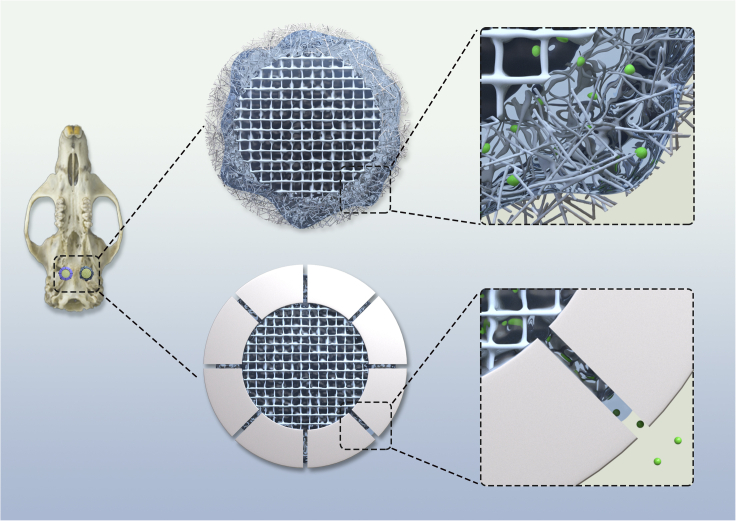
A schematic diagram of the core-shell scaffold for sustained release of GF

Here, we introduced the core-shell scaffold as a drug delivery platform to achieve sustained release of BMPs. The scaffold consists of two parts: the inner core and outer shell. The inner core can be a collagen scaffold, a super-absorbent sponge, or a hydrogel. The inner core absorbs the drug solution and stores it. The outer shell comprises dense fibers or a solid shell with microchannels (Figure 4) that diffuse the drug solution into the environment only through capillary action to delay the drug release. The solution can fill the core by capillary action by immersing the scaffold in the BMP aqueous solution. The structure and material composition of the inner core can be adjusted to regulate BMP loading. After implantation, the loaded BMP can be released into the injured area by capillary action through the outer shell. The density of the fiber shell or the number and diameter of the microchannels can be tuned to control the release rate of BMP.

#### Oral bolus to deliver insulin to the esophagus

The oral form of peptide and protein drugs (such as insulin) is a life-changing solution for patients who need multiple daily injections(Han et al., 2020). However, the GI tract is not ideal for processing large-molecule drugs. It is ultra-acidic (pH = 1–3) and full of the microbiome and digestive enzymes that can destabilize the formulation and denature/degrade peptide and protein drugs. Here, we introduced an oral bolus that encapsulates insulin or other peptide and protein drugs through the capillary action to improve drug availability of the oral drug.

**Figure 5:**
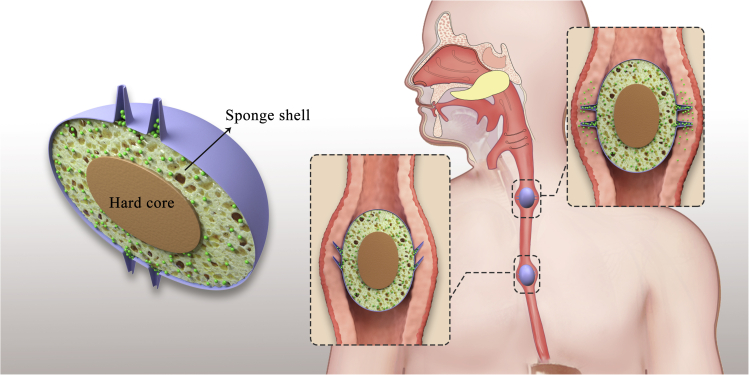
A schematic diagram of the bolus to deliver peptide and protein drugs to the esophagus The bolus has three parts: hardcore, sponge shell, and microneedles. The sponge shell stores the drugs in the bolus. When the bolus passes the esophagus, the bolus is compressed and releases peptide and protein drugs into the esophagus through the microneedles.

The bolus consists of three parts: a hardcore, a sponge shell, and multiple hollow microneedles (Figure 5). The hardcore is located in the center of the bolus and surrounded by the sponge shell. The sponge shell can store peptide and protein drug solutions by capillary action. The hollow microneedles and the sponge shell are inter-connected. Unlike the reported pill with microneedles that can tolerate the GI tract environment and deliver insulin to the lining of the small intestine and stomach (Abramson et al., 2019a, 2019b), the bolus is designed to deliver the peptide and protein drugs to the esophagus to avoid obstacles in the GI tract. Peristalsis moves bolus through the esophagus; the bolus is compressed, forcing the microneedles to penetrate the mucus layer. Simultaneously, the sponge shell is compressed by the hardcore and esophagus muscle, and the drug solution is injected into the mucus layer through the microneedles. The bolus can be football-shaped and only have microneedles in the middle to ensure all microneedles contact the mucus layer, avoiding mistargeting drugs. In addition, the microneedles are elastic, so when the bolus advances the esophagus, the microneedles repeatedly insert and pull out the mucus layer without straining the muscle tissue.

#### Semi-hollow floating ball for intravesical and gastroprotective drug delivery

A controlled drug delivery system with prolonged residence time in the stomach and bladder is of great practical importance for drug absorption. Floating microspheres-controlled drug delivery systems achieve long-term gastric retention up to 24 hr or longer(Bhadouriya et al., 2011; Zhao et al., 2021). On the other hand, the drug release from the floating microspheres is required to last for 24 hr to take full advantage of the long retention period. To achieve both long-term retention and sustained drug release, a semi-hollow floating ball was introduced.

**Figure 6:**
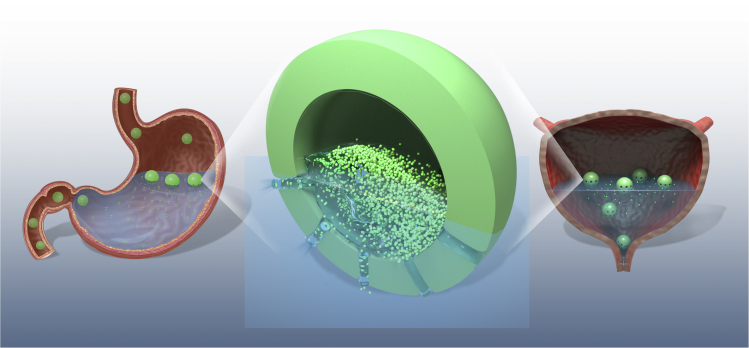
A schematic diagram of the semi-hollow floating ball for intravesical and gastroprotective drug delivery

The semi-hollow floating ball features as only one part of the ball is filled with solid medicine, and the other part empty (Figure 6). This structure allows the ball to float in the gastric juice in the stomach and the urine in the bladder to achieve long retention. In addition, the drug-filled part of the ball will always face the gastric juice or urine, and this part of the ball has channels through which liquid can enter the ball by capillary action. The liquid will dissolve the drugs in the ball and then flow out through the channels, achieving slow drug release. The liquid flowing rate of the ball can be restricted by adjusting the number and diameter of the channels. After the drug is completely dissolved, the ball is filled with fluid, sinks, and expels from the stomach and bladder.

## Conclusion and outlook

Drug delivery technology was developed in the 1950s. Tremendous progress has been made in this field, evidenced by the increasing number of publications, clinical trials, and medical practices related to drug delivery systems. Despite the progress made, it still has broad room for development. Innovative drug delivery strategies are still desired because (i) the existing drug delivery technologies face several challenges, such as biocompatibility concerns, low drug loading efficiency, unsatisfactory drug release control, and low drug bioavailability; (ii) existing clinical needs have not yet been met, with a newly emerging clinical need daily, such as controlling the COVID-19 pandemic; (iii) with the continuous development of innovative drugs, new therapies (such as cell therapy and gene therapy), and genetic engineering, case-specific drug delivery systems are needed to adapt to their progress.

Our review explored capillary action as a new drug delivery strategy. Capillary action is the interaction between liquid and material, which describes the ability of the liquid to stay and flow in a narrow space without the assistance of external forces. Contrary to the simplicity and popularity in our daily life, the role of capillary action in drug delivery has not attracted people's attention. Therefore, its role has not been actively developed. The basic principle of capillary action in drug delivery is that the material can hold the liquid by capillary action, so the drug dispersed or dissolved in the liquid can be encapsulated in the materials. The materials can transfer liquid on the surface so that they can deliver and release drugs. Many factors affect the capillary action's performance in drug encapsulation efficiency and release kinetics, such as the surface-to-volume ratio, surface polarity, nature of the liquid, and temperature. Understanding these factors and their effects can help maximize their role in drug delivery. By adjusting these factors, the drug loading capacity (how the liquid is absorbed into the material) and drug release profile (the speed at which the liquid passes through the material) of the drug delivery system can be regulated to meet the actual needs of specific disease treatment.

We introduced five prospective designs of drug delivery platforms based on capillary action: marker pen for topical and transdermal drug delivery, microneedle patch with a sponge container for pulsatile drug delivery, core-shell scaffold for sustained release of GF, oral bolus for insulin delivery to the esophagus, and semi-hollow floating ball for intravesical and gastroprotective drug delivery. As shown by these platforms, capillary action exhibits many unique characteristics that existing drug delivery technologies cannot easily achieve. Therefore, capillary action can solve some limitations of existing drug delivery systems, such as (i) achieve a high drug encapsulation with almost no drug wastage; (ii) achieve zero-order, pulsatile, and on-demand drug release; (iii) increase the bioavailability of drugs; (iv) can be green with no issue of the solvent residue because the process of drug loading and drug release does not involve organic solvents. These five platforms can be used to treat different diseases, such as pain management, skin infection treatment, heart regeneration, bond repair, and diabetes medication. As people pay more attention to the application of capillary action in drug delivery, many other drug delivery platforms based on capillary action are expected to be developed with unique functions. Capillary action could become an important choice for designing drug delivery systems to improve therapeutic drug efficacy, treat diseases, and ultimately improve human health.

Capillary action combined with other drug delivery strategies is suitable for water-soluble and poorly water-soluble drugs. For example, poorly water-soluble drugs can be covalently conjugated with hydrophilic polymers to improve their water affinity. The synthesized prodrug can be loaded into the drug delivery systems by capillary action. Drugs can be formulated into polymeric or inorganic particles through non-covalent interactions and cavity loading. When the obtained particles are uniformly dispersed in water, the drug therein can be encapsulated and released by capillary action.

As interdisciplinary research, the development of new drug delivery strategies requires new molecules, technologies to manufacture these molecules into new materials with designed structures, and corresponding characterization methods for evaluating the structure and function of the molecules and materials. Therefore, the development of drug delivery technology will promote the development of materials science, chemistry, chemical engineering, biomedical engineering, and other related fields.

### Limitations of the study

The focus of this paper is to introduce the principle of capillary action in drug encapsulation and controlled release and the advantages of capillary action over existing drug delivery strategies. Five capillary action-based drug delivery platforms are presented to illustrate the principle and potential applications of capillary action in drug delivery, medicine, and disease treatment. The authors would like to clarify that the capillary action-based drug delivery platforms are in the conceptual design stage. The manufacturing and optimization of each drug delivery platform require molecular design, material engineering, and pathological considerations, and can be a separate study.
